# Promoting mitochondrial dynamics by inhibiting the PINK1–PRKN pathway to relieve diabetic nephropathy

**DOI:** 10.1242/dmm.050471

**Published:** 2024-05-01

**Authors:** Jun-yi Zhu, Joyce van de Leemput, Zhe Han

**Affiliations:** ^1^ Center for Precision Disease Modeling, Department of Medicine, University of Maryland School of Medicine, Baltimore, MD 21201, USA; ^2^ Division of Endocrinology, Diabetes, and Nutrition, Department of Medicine, University of Maryland School of Medicine, Baltimore, MD 21201, USA

**Keywords:** Diabetes, Nephrocyte, *Drosophila*, Mitochondria, PINK1, PRKN

## Abstract

Diabetes is a metabolic disorder characterized by high blood glucose levels and is a leading cause of kidney disease. Diabetic nephropathy has been attributed to dysfunctional mitochondria. However, many questions remain about the exact mechanism. The structure, function and molecular pathways are highly conserved between mammalian podocytes and *Drosophila* nephrocytes; therefore, we used flies on a high-sucrose diet to model type 2 diabetic nephropathy. The nephrocytes from flies on a high-sucrose diet showed a significant functional decline and decreased cell size, associated with a shortened lifespan. Structurally, the nephrocyte filtration structure, known as the slit diaphragm, was disorganized. At the cellular level, we found altered mitochondrial dynamics and dysfunctional mitochondria. Regulating mitochondrial dynamics by either genetic modification of the Pink1–Park (mammalian PINK1–PRKN) pathway or treatment with BGP-15, mitigated the mitochondrial defects and nephrocyte functional decline. These findings support a role for Pink1–Park-mediated mitophagy and associated control of mitochondrial dynamics in diabetic nephropathy, and demonstrate that targeting this pathway might provide therapeutic benefits for type 2 diabetic nephropathy.

## Teaser

**Summary:** Using a *Drosophila* high-sucrose diet model, the study shows that a disrupted PINK1–PRKN pathway underlies the dysfunctional mitochondrial dynamics in diabetic nephropathy and demonstrates a potential therapeutic intervention.

## INTRODUCTION

Diabetic nephropathy is the most common cause of chronic kidney disease and end-stage renal failure globally. It is characterized by pathological quantities of urine albumin excretion, diabetic glomerular lesions and loss of glomerular filtration rate (Fineberg et al., 2013; Lim, 2014). Podocytes are glomerular epithelial cells located on the surface of the glomeruli capillaries that form the slit membrane, which filters the blood to prevent proteinuria (Reidy et al., 2014; Sugita et al., 2021). A dramatic decrease in podocyte number is observed at the early stage of diabetic nephropathy, resulting in the loss of filtration barrier integrity, glomerulosclerosis and ultimately renal failure (Qian et al., 2008; Wolf et al., 2005). Thus, podocyte injury is considered a major contributor to diabetic nephropathy (Ilatovskaya et al., 2015; Liu et al., 2022). Excess dietary sugar intake has been shown to cause metabolic disease, diabetes, obesity and hypertension, among others (Akinyanju et al., 1968; Bray et al., 2004; Faeh et al., 2005; Gross et al., 2004; Israel et al., 1983; Lee et al., 2022; Malik et al., 2010; Raben et al., 2002; Semnani-Azad et al., 2020). Indeed, the global rise in dietary sugar intake coincides with increased incidence of diabetic nephropathy (Gross et al., 2004; Malik et al., 2010).

The *Drosophila melanogaster* pericardial nephrocyte (hereafter, nephrocyte) bears striking structural and functional similarities to the mammalian podocyte (Fu et al., 2017; Na and Cagan, 2013; Simons and Huber, 2009; Weavers et al., 2009). Both nephrocytes and podocytes form highly specialized filtration structures known as slit diaphragms, which, together with the basement membrane, serve as size- and charge-dependent filtration barriers (Weavers et al., 2009). These similarities also apply to phenotypes in response to stress. For example, as for excess dietary sugars in humans, *Drosophila* nephrocytes in animals fed chronic high dietary sucrose, display defects that phenocopy aspects of diabetic nephropathy, including hyperglycemia, hyperlipidemia and insulin resistance (Kim et al., 2021; Na et al., 2015; Rani and Gautam, 2018). Furthermore, chronic dietary sucrose treatment induces morphological abnormalities in the mitochondria in the fly nephrocytes (Kim et al., 2021). The fly nephrocyte model has also been used to identify an effective treatment for nephrotic syndrome in patients caused by a specific *COQ2* variant (Zhu et al., 2017). Dietary supplementation with coenzyme Q_10_, the product of the CoQ pathway, of flies with nephrocyte-specific silencing of *Coq2* restored renal function decline, mitochondrial dysfunction and abnormal localization of the slit diaphragms (Zhu et al., 2017). In addition, *Drosophila* provides a low-cost, high-efficiency drug testing platform (Maitra and Ciesla, 2019; Su, 2019). These studies demonstrate the value of the *Drosophila* nephrocyte as a model system to study both the underlying mechanisms and possible treatments for diabetic nephropathy.

Like in other cells, in nephrocytes, the mitochondrion is the primary source of cellular ATP production. To maintain mitochondrion health, quality control and selective removal of damaged mitochondria are tremendously important (Tatsuta and Langer, 2008). The concept of mitochondrial quality control in the form of mitophagy has gained momentum over the past few years with the identification of the PTEN-induced kinase 1 (PINK1)–Parkin RBR E3 ubiquitin protein ligase (PRKN) pathway (Deng et al., 2008; Guo, 2012). PINK1–PRKN can regulate mitophagy to remove any damaged mitochondria. (Youle and van der Bliek, 2012). Mitochondrial dysfunction in podocytes has been observed in diabetic nephropathy and is associated with decreased mitochondrial membrane potential (Qi et al., 2017). Moreover, high glucose in the culture medium promotes mitochondrial fragmentation in retinal vascular cells (Roy et al., 2019). This has been attributed to reduced mitofusin 2 (Mfn2) protein expression, which impairs mitochondrial fusion in diabetes (Dai and Jiang, 2019; Williams and Caino, 2018). However, studies in animal models have also reported enlarged mitochondria in response to a high-sucrose diet (Kaneda et al., 1992; Kim et al., 2021). As such, much remains unknown about the mitochondrial dynamics and dysfunction associated with diabetic nephropathy.

Here, we used *Drosophila* as an *in vivo* model system to investigate diabetic nephropathy. *Drosophila* with dietary high-sucrose treatment showed significant levels of nephrocyte functional decline, decreased cell size, shortened lifespan and mitochondrial dysfunction associated with mitochondrial fission defects. Regulating mitochondrial dynamic control by genetically modifying the Pink1–Park (human PINK1–PRKN) pathway or treatment with the drug BGP-15 attenuated the mitochondrial dysfunction and renal functional decline in the flies.

## RESULTS

### Dietary high-sucrose treatment in *Drosophila* causes nephrocyte functional decline, decreased cell size and a shortened lifespan

**Fig. 1.**
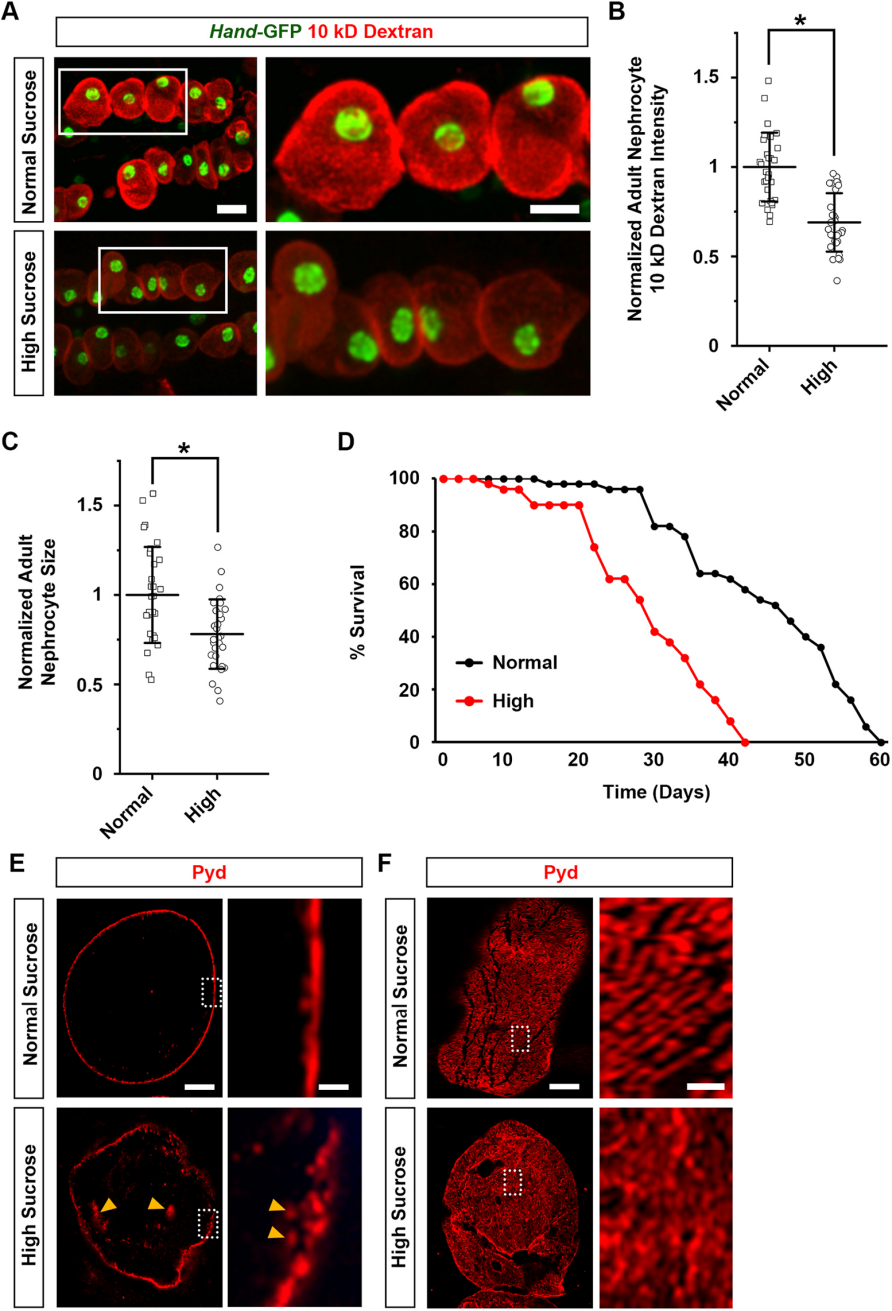
**High-sucrose treatment reduces nephrocyte function, decreases cell size, shortens *Drosophila* lifespan and disrupts nephrocyte slit diaphragm structure.** (A) 10 kDa dextran uptake (red) by nephrocytes from flies (4-day-old adult females) on a normal diet (normal sucrose) and those treated with a high-sucrose diet. *Hand*-GFP transgene expression was visualized as green fluorescence concentrated in the nephrocyte nuclei (*Hand*-GFP;*Dot*-Gal4 flies). Scale bars: 25 µm (left images); 15 µm (magnifications of boxed area). (B) Quantification of 10 kDa dextran uptake by nephrocytes from high-sucrose-treated flies relative to that in flies on a normal diet. *n*=30 nephrocytes from six flies per group (4-day-old adult females; *Hand*-GFP;*Dot*-Gal4). Results show mean±s.d. normalized to the control group. *P<0.05 [Shapiro–Wilk test (α=0.05) indicated normal distribution; two-tailed unpaired Student's *t*-test, *P*=7.5×10^−8^]. (C) Quantification of nephrocyte cell size from high-sucrose-treated flies relative to that in flies on a normal diet. *n*=30 nephrocytes from six flies per group (4-day-old adult females; *Hand*-GFP;*Dot*-Gal4). Results show mean±s.d. normalized to the control group. *P<0.05 [Shapiro–Wilk test (α=0.05) indicated normal distribution; two-tailed unpaired Student's *t*-test, *P*=0.00112]. (D) Adult survival curves for flies on a normal diet and those treated with a high-sucrose diet. *n*=100 flies per group (males; *Hand*-GFP;*Dot*-Gal4). (E,F) Localization of slit diaphragm protein polychaetoid (Pyd; red) by immunofluorescence in nephrocytes from normal diet and high-sucrose-treated flies (4-day-old adult females; *Dot*>mito-GFP). (E) Images at the nephrocyte medial optical section. Orange arrowheads indicate internalized Pyd. (F) For E,F, each experiment was conducted twice, each including four flies per genotype. For each fly, images from three nephrocytes were acquired. Scale bars: 5 µm (left images); 1 µm (magnification of boxed area).

Previous research has demonstrated that high-sucrose treatment for *Drosophila* leads to a phenotype reminiscent of diabetes, including hyperglycemia, hyperlipidemia and insulin resistance (Na et al., 2015). Here, we treated the flies with 0.2 g/ml sucrose (i.e. a high-sucrose diet), and then studied the effect on the nephrocytes using assays we developed previously (Zhang et al., 2013). To determine the effect on function, we used an *ex vivo* assay that measures the capacity of dissected nephrocytes to filter and endocytose 10 kDa fluorescent dextran particles. We observed a significant reduction of 10 kDa dextran intensity in nephrocytes from high-sucrose-treated flies compared to those from flies fed a normal sucrose diet (Fig. 1A,B). In addition, nephrocyte size was significantly reduced following high-sucrose treatment (Fig. 1C), and high-sucrose treatment led to a shortened lifespan compared to that in flies consuming normal sucrose food (Fig. 1D). These findings indicate high sucrose induces nephrocyte size and functional defects that negatively affect fly viability.

### High-sucrose treatment in *Drosophila* affects the slit diaphragm filtration structure

The slit diaphragm structure is essential for the nephrocyte filtration function (van de Leemput et al., 2022; Wen et al., 2020). To examine its structural integrity in nephrocytes after high-sucrose treatment, we carried out immunochemistry for the slit diaphragm protein Polychaetoid (Pyd) (mammalian tight junction protein 1, TJP1, also known as ZO-1). Localization of Pyd in the medial optical section of nephrocytes showed a fine and continuously delineated circumferential ring in flies consuming normal sucrose food (Fig. 1E). On the surface of these nephrocytes, Pyd presented a uniform and smoothly distributed fingerprint-like localization pattern (Fig. 1F). High-sucrose treatment disrupted nephrocyte Pyd localization, such that much Pyd protein was no longer at the surface but was internalized (Fig. 1E, arrowheads). High-sucrose-treated nephrocytes also showed signs of a severely disrupted slit diaphragm fingerprint-like localization pattern (Fig. 1F). The results imply that structural disruption of the slit diaphragm causes nephrocyte dysfunction following a high-sucrose diet.

### High-sucrose treatment in *Drosophila* disrupts mitochondria dynamics and function

**Fig. 2.**
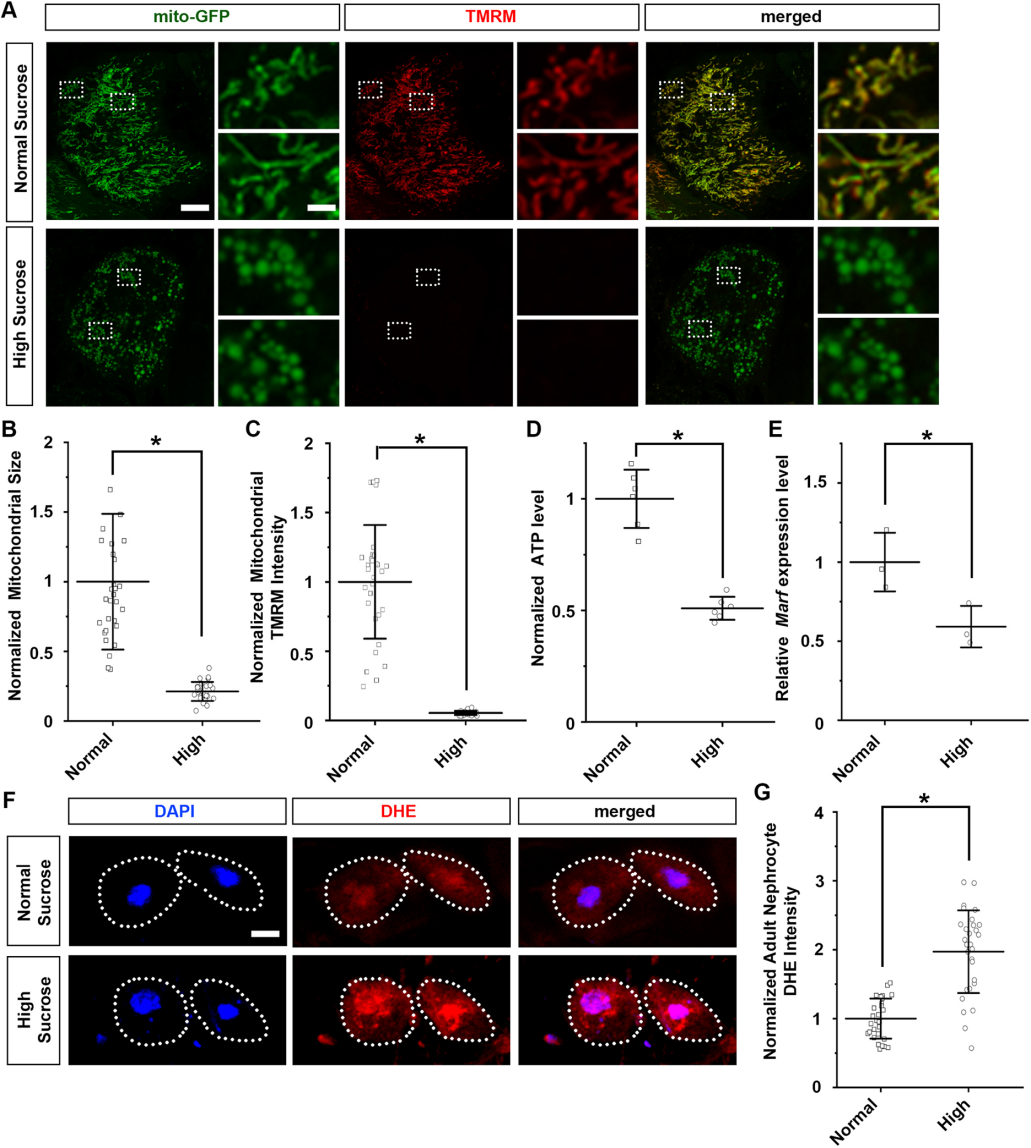
**High-sucrose treatment causes mitochondrial dysfunction and increased levels of ROS.** (A) Mitochondrial morphology in nephrocytes from normal diet and high-sucrose-treated flies. UAS-mito-GFP induced by nephrocyte-specific driver *Dot*-Gal4 labels the mitochondria (*Dot*>mito-GFP). Mitochondrial membrane potential was detected through TMRM fluorescence. Scale bars: 5 µm (left images); 1 µm (magnification of boxed area). (B) Quantification of mitochondrial size in nephrocytes from high-sucrose-treated flies relative to that in flies on a normal diet. *n*=size 30 mitochondria per nephrocyte averaged, for 30 nephrocytes from six flies per dietary group (4-day-old adult females; *Dot*>mito-GFP). Results show mean±s.d. normalized to the control group. *P<0.05 [Shapiro–Wilk test (α=0.05) indicated normal sucrose condition not normally distributed; Mann–Whitney *U*-test, *P*=3.3×10^−11^]. (C) Quantification of mitochondrial TMRM fluorescence intensity in nephrocytes from high-sucrose-treated flies relative to that in flies on a normal diet. *n*=mitochondria in 30 nephrocytes from six flies per group (4-day-old adult females; *Dot*>mito-GFP). Results show mean±s.d. normalized to the control group. *P<0.05 [Shapiro–Wilk test (α=0.05) indicated normal distribution; two-tailed unpaired Student's *t*-test, *P*=3.71×10^−13^]. (D) Quantification of ATP level for high-sucrose-treated flies relative to that in flies on a normal diet. *n*=6 flies per group (4-day-old adult females; *Dot*>mito-GFP). Results show mean±s.d. normalized to the control group. *P<0.05 [Shapiro–Wilk test (α=0.05) indicated normal distribution; two-tailed unpaired Student's *t*-test, *P*=8.11×10^−5^]. (E) Quantification of *Marf* expression in nephrocytes for high-sucrose-treated flies relative to that in flies on a normal diet. *n*=50 adult flies per group (4-day-old adult females; *Dot*>mito-GFP); three biological repeats. Results show mean±s.d. normalized to the control group. *P<0.05 [Shapiro–Wilk test (α=0.05) indicated normal distribution; two-tailed unpaired Student's *t*-test, *P*=0.035]. (F) Levels of ROS in nephrocytes from normal diet and high-sucrose-treated flies. DAPI was used to visualize the nucleus. Dotted line indicates cell boundary. ROS levels were detected through DHE fluorescence (red). Scale bar: 5 µm. (G) Quantification of DHE fluorescent intensity in nephrocytes from high-sucrose-treated flies relative to that in flies on a normal diet. *n*=30 nephrocytes from six flies per group (4-day-old adult females; *Dot*>mito-GFP). Results show mean±s.d. normalized to the control group. *P<0.05 [Shapiro–Wilk test (α=0.05) indicated normal distribution; two-tailed unpaired Student's *t*-test, *P*=2.45×10^−9^].

Mitochondria function is highly dependent on dynamic morphological changes in size and shape, directed by fission and fusion (de Boer et al., 2022). To investigate the effect of high-sucrose diet on the role of mitochondrial dynamics in nephrocytes, we employed the UAS-GAL4 system combined with UAS-mito-GFP, which specifically labels the mitochondria to visualize their morphology. We found that in nephrocytes from flies consuming normal sucrose food, the mitochondria showed their typical round shape (Fig. 2A, magnified image, top). We also observed areas with elongated mitochondria morphology, indicative of mitochondria dynamics (Fig. 2A, magnified image, bottom). However, with high-sucrose treatment, the mitochondria in the nephrocytes showed a significantly reduced size and a reduced capacity to change their morphology, indicative of aberrant mitochondrial fission–fusion (Fig. 2A,B). Moreover, under high-sucrose conditions, the expression of *Mitochondrial assembly regulatory factor* (*Marf*), the *Drosophila* homolog of human *MFN2*, was significantly reduced (Fig. 2E), supporting defective fusion.

To detect any effect on mitochondria activity, we used the fluorescent dye tetramethylrhodamine, methyl ester (TMRM), an indicator of mitochondrial membrane potential (Perry et al., 2011). The mitochondria in nephrocytes from flies treated with high sucrose showed significantly reduced membrane potential, to barely detectable levels (Fig. 2A,C). In addition, ATP production in these mitochondria was significantly reduced (Fig. 2D), whereas the levels of reactive oxygen species (ROS), were significantly increased [observed as increased dihydroethidium (DHE) signal] (Fig. 2F,G). An uncontrolled rise in ROS can be detrimental to the cell, by activating inflammation, ultimately leading to apoptosis (Finkel, 2012).

These data demonstrate that a high-sucrose diet induces both structural and functional mitochondria defects, which disrupt mitochondrial dynamics in the nephroctyes.

### Pink1–Park-mediated control of mitochondrial dynamics in *Drosophila* nephrocytes

Mitochondrial dynamics are in part controlled through the Pink1–Park pathway, which regulates mitophagy to remove damaged mitochondria (Guo, 2012). Therefore, we next examined whether genetic modification of the Pink1–Park pathway in *Drosophila* nephrocytes affected mitochondrial morphology and function. To achieve this, we combined the nephrocyte-specific driver *Dot*-Gal4 with either overexpression (UAS-*park*-OE or UAS-*Pink1*-OE) or RNAi knockdown (UAS-*park*-RNAi or UAS-*Pink1*-RNAi) of *park* or *Pink1* in flies. The progenies of the *Dot*-Gal4 with UAS crosses carried the nephrocyte-specific expression of Gal4, the driver for either the targeted overexpression or silencing of *park* or *Pink1*. Two independent RNAi lines were studied for each gene. These provided the same results; therefore, representative data for one line have been displayed in the figures.

**Fig. 3.**
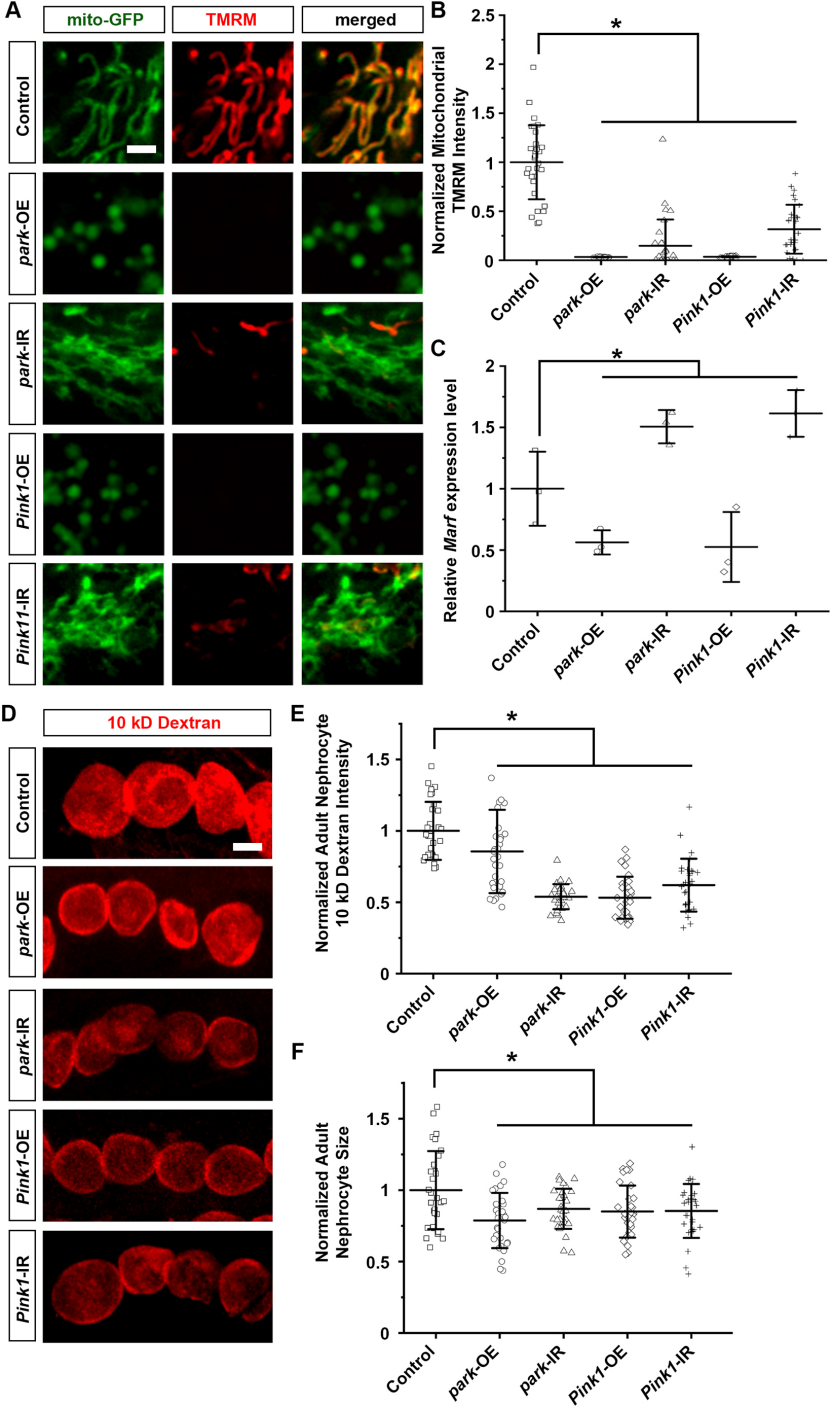
**Pink1–Park pathway interference leads to altered nephrocyte mitochondrial morphology and reduced nephrocyte function.** (A–F) Flies used (4-day-old adult females) were: control (*Dot*>mito-GFP); *park*-OE (*Dot*>mito-GFP,*park*-OE); *park*-IR (*Dot*>mito-GFP,*park*-RNAi); Pink1-OE (*Dot*>mito-GFP,*Pink1*-OE); Pink1-IR (*Dot*>mito-GFP,*Pink1*-RNAi). (A) Mitochondrial morphology visualized by UAS-mito-GFP induced by nephrocyte-specific driver *Dot*-Gal4 (*Dot*>mito-GFP). Mitochondrial membrane potential detected through TMRM fluorescence. Scale bar: 1 µm. (B) Quantification of mitochondrial TMRM intensity in nephrocytes that overexpressed or inhibited *park* or *Pink1* relative to control nephrocytes (*Dot*>mito-GFP). *n*=mitochondria in 30 nephrocytes from six flies per group. Results show mean±s.d. normalized to the control group. *P<0.05 [Shapiro–Wilk test (α=0.05) indicated *park*-IR data not normally distributed; Kruskal–Wallis H-test followed by Dunn's test; control versus *park*-OE, *P*=3.01×10^−11^; control versus *park*-IR, *P*=1.38×10^−9^; control versus *Pink1*-OE, *P*=3.02×10^−11^; control versus *Pink1*-IR, *P*=9.26×10^−9^]. (C) Quantification of *Marf* expression in nephrocytes that overexpressed or inhibited *park* or *Pink1* relative to that in control nephrocytes (*Dot*>mito-GFP). *n*=50 adult flies per group; three biological repeats. Results show mean±s.d. normalized to the control group. *P<0.05 [Shapiro–Wilk test (α=0.05) indicated normal distribution; one-way ANOVA followed by Tukey-Kramer post-test; control versus *park*-OE, *P*=0.017; control versus *park*-IR, *P*=0.021; control versus *Pink1*-OE, *P*=0.029; control versus *Pink1*-IR, *P*=0.0365]. (D) 10 kDa dextran uptake (red) by nephrocytes. Scale bar: 25 µm. (E) Quantification of 10 kDa dextran uptake by nephrocytes that overexpressed or inhibited *park* or *Pink1* relative to control nephrocytes. *n*=30 nephrocytes from six flies per group. Results show mean±s.d. normalized to the control group. *P<0.05 [Shapiro–Wilk test (α=0.05) indicated *park*-OE and *Pink1*-OE data not normally distributed; Kruskal–Wallis H-test followed by Dunn's test; control versus *park*-OE, *P*=0.01628; control versus *park*-IR, *P*=4.97×10^−11^; control versus *Pink1*-OE, *P*=2.61×10^−10^; control versus *Pink1*-IR, *P*=5.46×10^−9^]. (F) Quantification of nephrocyte cell size that overexpress or inhibit *park* or *Pink1* relative to control nephrocytes. *n*=30 nephrocytes from six flies per group. Results show mean±s.d. normalized to the control group. *P<0.05 [Shapiro–Wilk test (α=0.05) indicated normal distribution; one-way ANOVA followed by Tukey-Kramer post-test; control versus *park*-OE, *P*=0.000393; control versus *park*-IR, *P*=0.05383; control versus *Pink1*-OE, *P*=0.0309; control versus *Pink1*-IR, *P*=0.03926].

Overexpressing *park* or *Pink1* in the nephrocytes dramatically reduced mitochondrial size and changed mitochondrial morphology in these cells, indicative of aberrant mitochondrial fission–fusion (Fig. 3A). These mitochondria morphological changes coincided with significantly reduced *Marf* levels (Fig. 3C), which is known to be regulated by Pink1–Park (Yun et al., 2014; Ziviani et al., 2010). TMRM was absent in these mitochondria, indicating significantly reduced membrane potential (Fig. 3A,B). These mitochondrial phenotypes are similar to those observed in nephrocytes following high-sucrose treatment above (Fig. 2). By contrast, silencing *park* or *Pink1* enlarged mitochondrial size in the nephrocytes, a sign of altered mitochondrial fission–fusion dynamics (Fig. 3A). *Marf* levels were significantly increased in these nephrocytes (Fig. 3C). The membrane potential of their mitochondria was significantly reduced as shown by a reduced TMRM signal (Fig. 3A,B).

Next, we looked at changes in uptake function in the nephrocytes with altered *Pink1*–*park* expression. We observed a significant reduction of 10 kDa dextran intensity in nephrocytes following either *Pink1*–*park* pathway overexpression or silencing compared to that in nephrocytes from control flies (*Dot*-Gal4-driven mito-GFP), indicating a decline in nephrocyte uptake function (Fig. 3D,E). Notably, nephrocyte sizes were significantly reduced after *Pink1*–*park* pathway overexpression or silencing (Fig. 3F). These findings are reminiscent of the dysfunctional nephrocyte filtration we observed following a high-sucrose diet (Fig. 2).

Together, these data show that genetic modification of Pink1–Park-mediated mitophagy in nephrocytes affects mitochondrial dynamics, which leads to dysfunctional nephrocytes.

### Pink1–Park pathway inhibition attenuated the mitochondrial defects and nephrocyte functional decline caused by a high-sucrose diet

**Fig. 4.**
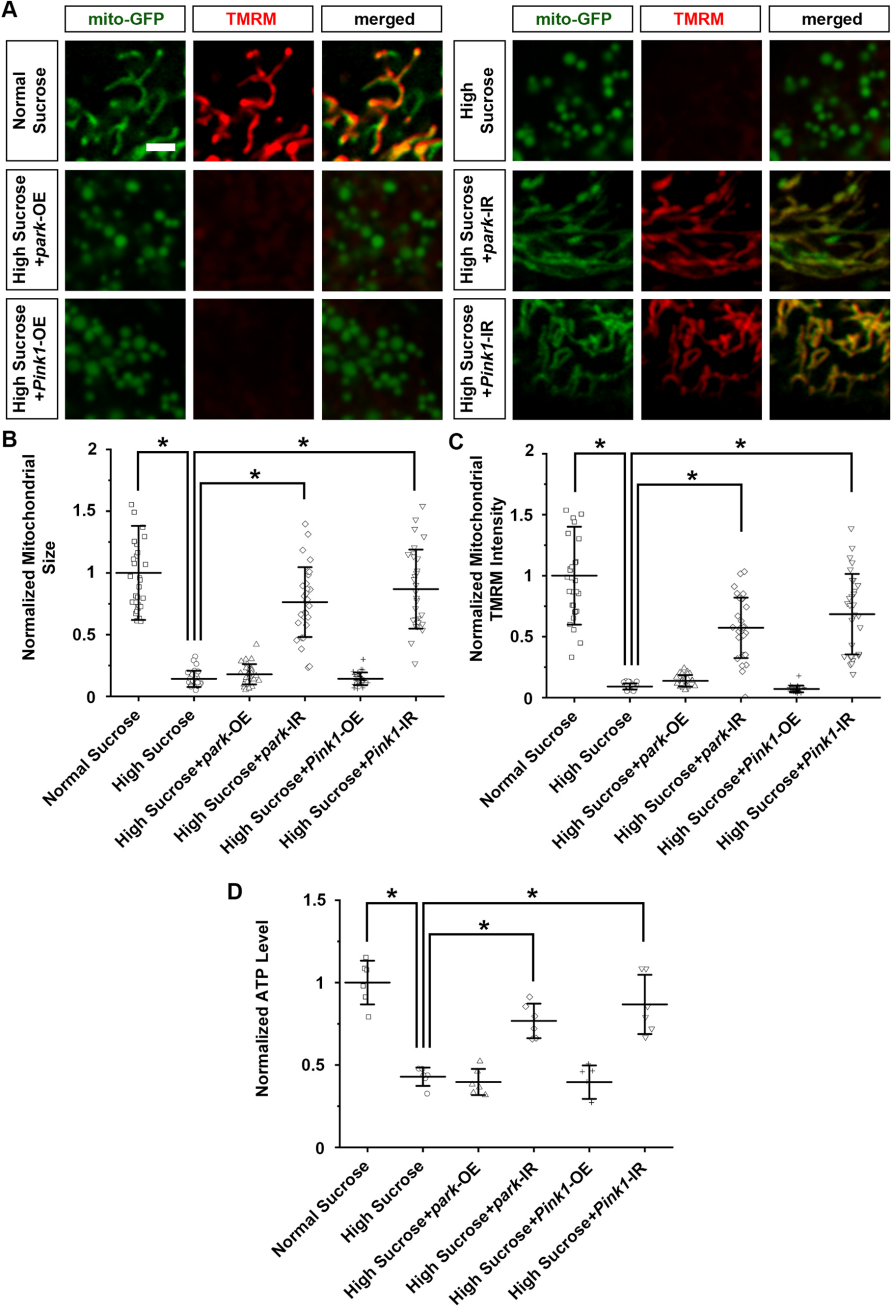
**Pink1–Park pathway inhibition attenuated nephrocyte mitochondrial morphological and functional defects.** (A–D) Flies used (4-day-old adult females): control (*Dot*>mito-GFP); *park*-OE (*Dot*>mito-GFP,*park*-OE); *park*-IR (*Dot*>mito-GFP,*park*-RNAi); Pink1-OE (*Dot*>mito-GFP,*Pink1*-OE); Pink1-IR (*Dot*>mito-GFP,*Pink1*-RNAi). (A) Mitochondrial morphology visualized by UAS-mito-GFP induced by nephrocyte-specific driver *Dot*-Gal4. Mitochondrial membrane potential detected through TMRM fluorescence. Scale bar: 1 µm. (B) Quantification of mitochondrial size in nephrocytes from flies on a high-sucrose diet and a normal sucrose control. *n*=size 30 mitochondria per nephrocyte averaged, for 30 nephrocytes from six flies per group. Results show mean±s.d. normalized to the control group. *P<0.05 [Shapiro–Wilk test (α=0.05) indicated high-sucrose data not normally distributed; Kruskal–Wallis H-test followed by Dunn's test; normal versus high, *P*=3.012×10^−11^; high versus high+*park*-IR, *P*=7.364×10^−10^; high versus high+*Pink1*-IR, *P*=3.681×10^−11^]. (C) Quantification of mitochondrial TMRM intensity in nephrocytes that overexpressed or inhibited *park* or *Pink1*, from flies on a high-sucrose diet relative to nephrocytes from control flies (*Dot*>mito-GFP) on a high-sucrose or normal sucrose diet. *n*=mitochondria in 30 nephrocytes from six flies per group. Results show mean±s.d. normalized to the control group. *P<0.05 [Shapiro–Wilk test (α=0.05) indicated normal sucrose and high-sucrose plus *Pink1*-OE data were not normally distributed; Kruskal–Wallis H-test followed by Dunn's test; normal versus high, *P*=3.02×10^−11^; high versus high+*park*-IR, *P*=5.5×10^−10^; high versus high+*Pink1*-IR, *P*=3.02×10^−11^]. (D) Quantification of ATP level in flies on a high-sucrose diet and a normal sucrose control. *n*=6 flies per group. Results show mean±s.d. normalized to the control group. *P<0.05 [Shapiro–Wilk test (α=0.05) indicated normal distribution; one-way ANOVA followed by Tukey–Kramer post-test; normal versus high, *P*=0.0001341; high versus high+*park*-IR, *P*=0.0003784; high versus high+*Pink1*-IR, *P*=0.0001346].

Next, we examined whether genetic modification of the Pink1–Park pathway in *Drosophila* nephrocytes could mitigate the mitochondrial morphological defects and dysfunction caused by high-sucrose treatment. Using the same approach as above, we overexpressed or knocked down *park* or *Pink1* with the nephrocyte-specific driver *Dot*-Gal4, however, this time in high-sucrose-treated flies. Overexpressing *park* or *Pink1* failed to make a difference, but silencing *park* or *Pink1* restored the mitochondria morphology and their membrane potential under high-sucrose conditions to within the normal range (Fig. 4A–C). In addition, silencing *park* or *Pink1*, but not their overexpression, attenuated the diminished ATP production caused by high-sucrose treatment (Fig. 4D).

**Fig. 5.**
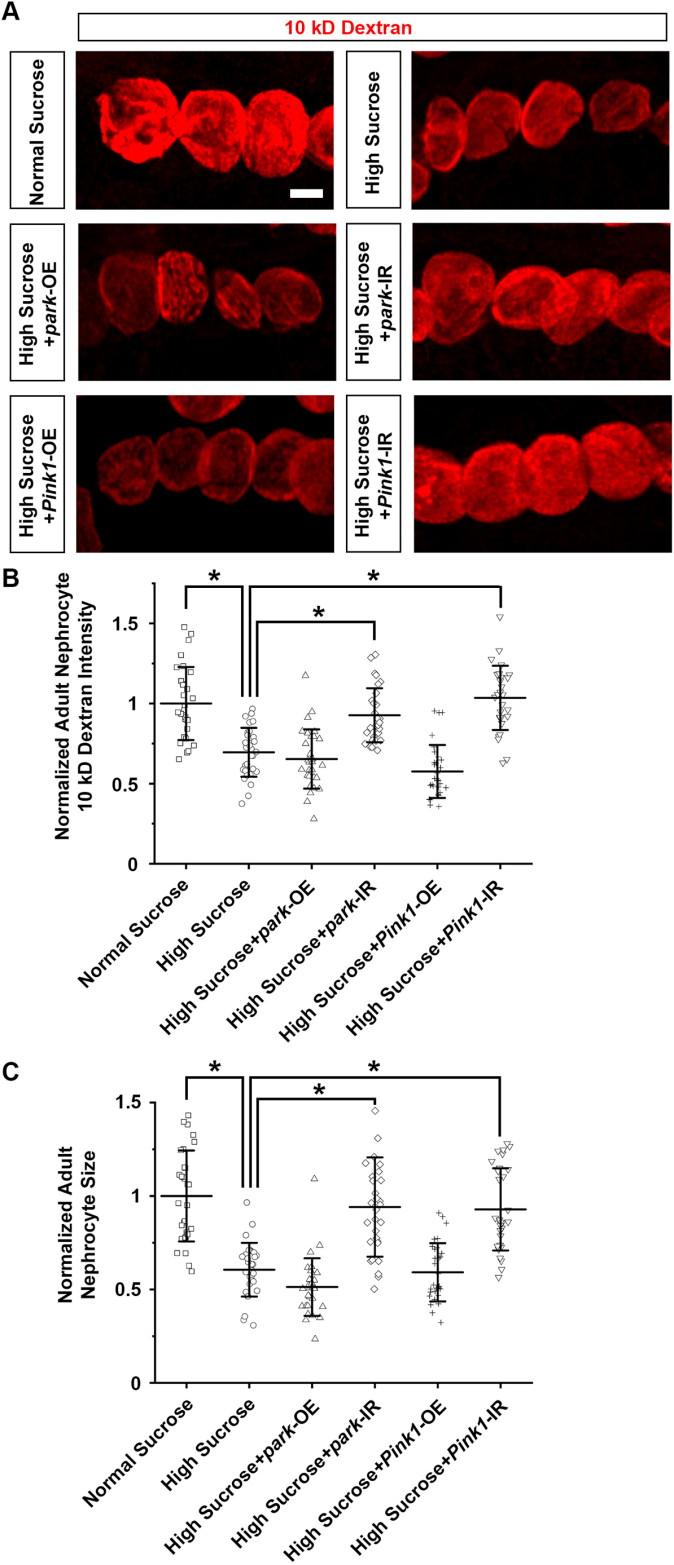
**Pink1–Park pathway inhibition attenuates nephrocyte functional decline and cell size reduction.** (A–C) Flies used (4-day-old adult females): control (*Dot*>mito-GFP); *park*-OE (*Dot*>mito-GFP,*park*-OE); *park*-IR (*Dot*>mito-GFP,*park*-RNAi); Pink1-OE (*Dot*>mito-GFP,*Pink1*-OE); Pink1-IR (*Dot*>mito-GFP,*Pink1*-RNAi). (A) 10 kDa dextran uptake (red) by nephrocytes from flies on a high-sucrose diet and a normal sucrose control. Scale bar: 25 µm. (B) Quantification of 10 kDa dextran uptake by nephrocytes. *n*=30 nephrocytes from six flies per group. Results show mean±s.d. normalized to the control group. *P<0.05 [Shapiro–Wilk test (α=0.05) indicated high-sucrose plus *park*-OE and high-sucrose plus *Pink1*-IR data were not normally distributed; Kruskal–Wallis H-test followed by Dunn's test; normal versus high, *P*=1.28×10^−6^; high versus high+*park*-IR, *P*=5.859×10^−6^; high versus high+*Pink1*-IR, *P*=4.686×10^−8^]. (C) Quantification of cell size for nephrocytes from flies on a high-sucrose diet and a normal sucrose control. *n*=30 nephrocytes from six flies per group. Results show mean±s.d. normalized to the control group. *P<0.05 [Shapiro–Wilk test (α=0.05) indicated high-sucrose plus *park*-OE and high-sucrose plus *Pink1*-IR data were not normally distributed; Kruskal–Wallis H-test followed by Dunn's test; normal versus high, *P*=1.429×10^−8^; high versus high+*park*-IR, *P*=8.841×10^−7^; high versus high+*Pink1*-IR, *P*=7.695×10^−8^].

Furthermore, the mitochondrial restoration in nephrocytes seen upon silencing *park* or *Pink1* under high-sucrose conditions significantly relieved the nephrocyte functional decline and cell size changes (Fig. 5A–C). This rescue was not observed in high-sucrose-treated flies with *park* or *Pink1* overexpression (Fig. 5A–C).

Taken together, these findings imply that dysfunctional Pink1–Park pathway-mediated mitophagy contributes to the high-sucrose-induced nephrocyte defects in flies.

### BGP-15 treatment attenuates the mitochondrial defects and nephrocyte functional decline caused by a high-sucrose diet

**Fig. 6.**
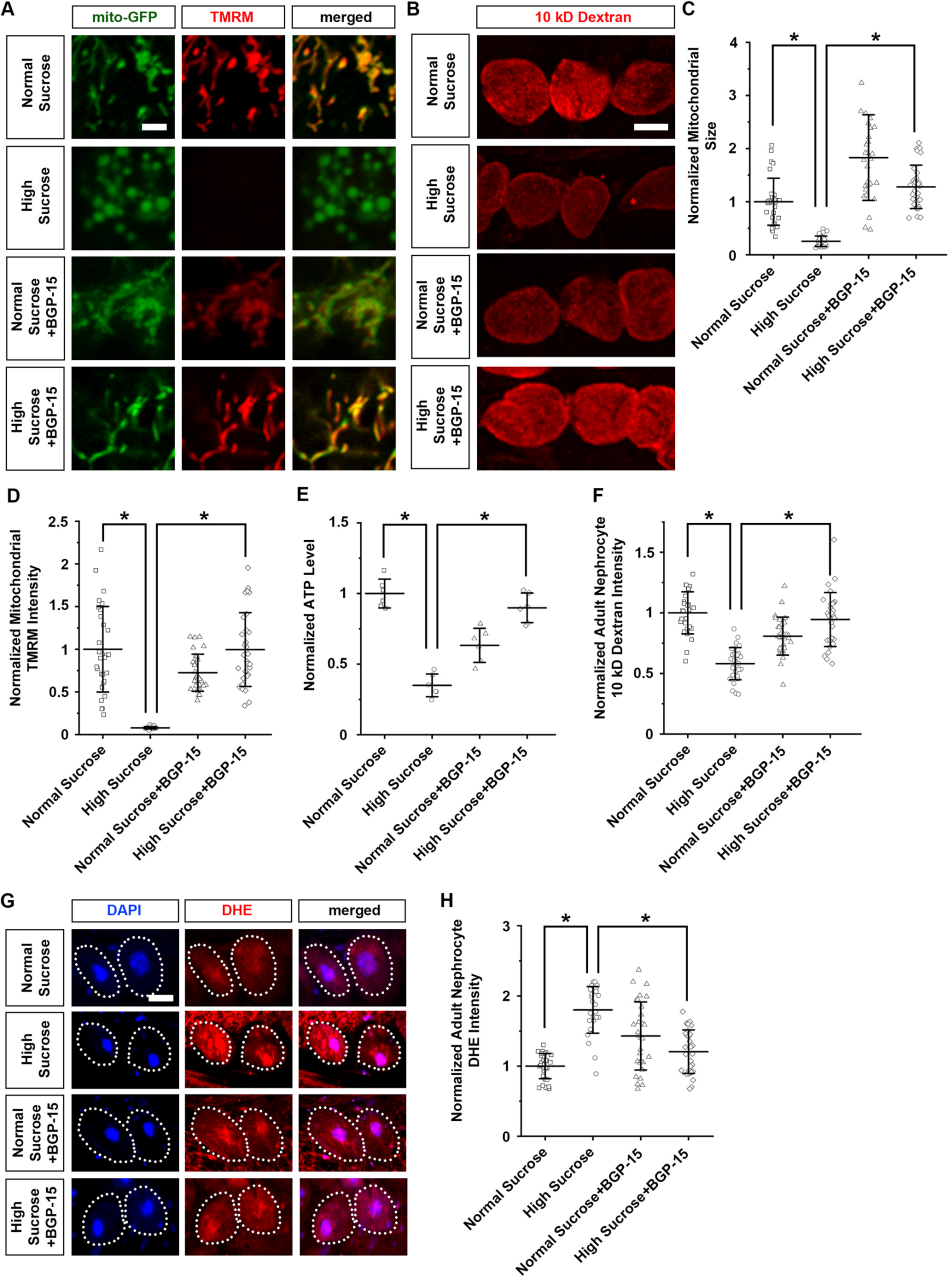
**BGP-15 treatment attenuates nephrocyte functional decline and cell size reduction.** (A) Mitochondrial morphology in nephrocytes from flies on a normal or a high-sucrose diet, with or without BGP-15 treatment (10 μM; from first-instar larva) (4-day-old adult females; *Dot*>mito-GFP). Mitochondrial membrane potential was detected through TMRM fluorescence (red). Scale bar: 1 µm. (B) 10 kDa dextran uptake (red) by nephrocytes from flies on a normal or a high-sucrose diet with or without BGP-15 treatment (10 μM; from first-instar larva) (4-day-old adult females; *Dot*>mito-GFP). Scale bar: 25 µm. (C) Quantification of mitochondrial size in nephrocytes from flies on a normal or a high-sucrose diet with or without BGP-15 treatment (10 μM; from first-instar larva) (4-day-old adult females; *Dot*>mito-GFP). Data presented relative to that from control flies on a normal diet. *n*=size 30 mitochondria per nephrocyte averaged for 30 nephrocytes from six flies per group. Results show mean±s.d. normalized to the control group. *P<0.05 [Shapiro–Wilk test (α=0.05) indicated normal sucrose and high-sucrose conditions were not normally distributed; Kruskal–Wallis H-test followed by Dunn's test; normal versus high, *P*=6.047×10^−11^; high versus high+BGP-15, *P*=3.01×10^−11^]. (D) Quantification of mitochondrial TMRM fluorescent intensity in nephrocytes from flies on a normal or a high-sucrose diet with or without BGP-15 treatment (10 μM; from first-instar larva) (4-day-old adult females; *Dot*>mito-GFP). Data presented relative to that from control flies on a normal diet. *n*=mitochondria in 30 nephrocytes from six flies per group. Results show mean±s.d. normalized to the control group. *P<0.05 [Shapiro–Wilk test (α=0.05) indicated high-sucrose and normal sucrose plus BGP-15 conditions were not normally distributed; Kruskal–Wallis H-test followed by Dunn's test; normal versus high, *P*=3.016×10^−11^; high versus high+BGP-15, *P*=0.009068]. (E) Quantification of ATP level in flies on a normal or a high-sucrose diet with or without BGP-15 treatment (10 μM; from first-instar larva); data presented relative to control flies on a normal diet. *n*=6 flies per group (4-day-old adult females; *Dot*>mito-GFP). Results show mean±s.d. normalized to the control group. *P<0.05 [Shapiro–Wilk test (α=0.05) indicated normal distribution; one-way ANOVA followed by Tukey-Kramer post-test; normal versus high, *P*=0.0001754; high versus high+BGP-15, *P*=0.0001754]. (F) Quantification of 10 kDa dextran uptake by nephrocytes of flies on a normal or high-sucrose diet with or without BGP-15 treatment (10 μM; from first-instar larva); data normalized to control flies on a normal diet. *n*=30 nephrocytes from six flies per group (4-day-adult females; *Dot*>mito-GFP). Results show mean±s.d. normalized to the control group. *P<0.05 [Shapiro–Wilk test (α=0.05) indicated normal distribution; one-way ANOVA followed by Tukey–Kramer post-test; normal versus high, *P*=0.0001367; high versus high+BGP-15, *P*=0.0001367]. (G) Levels of ROS in nephrocytes from flies on a normal or a high-sucrose diet with or without BGP-15 treatment (10 μM; from first-instar larva) (4-day-adult females; *Dot*>mito-GFP). DAPI was used to visualize the nucleus. Dotted line indicates cell boundary. Nephrocyte ROS levels detected through DHE fluorescence (red). Scale bar: 5 µm. (H) Quantification of DHE intensity in nephrocytes from flies (4-day-adult females; *Dot*>mito-GFP) on a normal or high-sucrose diet, with or without BGP-15 treatment (10 μM; from first-instar larva); data normalized to that for control flies on a normal diet. *n*=30 nephrocytes from six flies per group. Results show mean±s.d. normalized to the control group. *P<0.05 [Shapiro–Wilk test (α=0.05) indicated the high-sucrose condition was not normally distributed; Kruskal–Wallis H-test followed by Dunn's test; normal versus high, *P*=6.114×10^−10^; high versus high+BGP-15, *P*=8.345×10^−8^].

BGP-15 is a small molecule that acts on mitochondria quality control and protects against oxidative stress (Horvath et al., 2021; Sumegi et al., 2017). It has been reported to be safe and well tolerated, and was initially developed to treat insulin resistance (Literáti-Nagy et al., 2009; Pető et al., 2020). Therefore, we tested whether treating the flies on the high-sucrose diet with BGP-15 could attenuate their mitochondria functional and morphological defects. Starting at the first-instar larval stage, the flies were administered different doses of BGP-15 (0, 5, 10 and 20 μM) through their food. A 20 μM dose of BGP-15 was toxic to the flies, resulting in near-complete lethality across the high-sucrose-treated flies, with little effect on flies on a normal diet (Fig. S1). By contrast, a 5 μM dose of BGP-15 had no detectable effect on either normal diet or high-sucrose-treated flies (Fig. S1). Thus, for treatment we administered a 10 μM dose of BPG-15 to the flies; this significantly attenuated the mitochondrial morphological changes (Fig. 6A,C), the reduced membrane potential (Fig. 6A,D), the reduced ATP production (Fig. 6E) and the increased ROS (Fig. 6G,H), as well as the nephrocyte functional decline (Fig. 6B,F) associated with the high-sucrose diet. Taken together, these results further strengthen the link between mitochondrial dynamic control and diabetic nephropathy.

## DISCUSSION

The fly nephrocyte is a podocyte-like cell with slit diaphragm and lacunar channels for filtration (Weavers et al., 2009). Since this discovery over a decade ago, the *Drosophila* nephrocyte has grown into a valuable model to study kidney development and diseases (Dow et al., 2022; Rani and Gautam, 2018; van de Leemput et al., 2022). One such disease is diabetic nephropathy, for which the role of mitochondrial dynamics is of great interest (Gollmer et al., 2020; Sivitz and Yorek, 2010). Notably, the DRP1 (also known as DNM1L)–MFN2-mediated fission and fusion and the PINK1–PRKN-mediated mitophagy pathways that regulate mitochondrial dynamics to maintain its function are highly conserved from *Drosophila* to humans (Abtahi et al., 2020; Gegg et al., 2010; Guo, 2012). Mitochondria are highly dynamic, with constant fission and fusion to meet the energy demands of a cell and to respond to physiological stresses. DRP1 (fly Drp1) is a key component in promoting fission, during which the mitochondria are randomly divided (Germain, 2008). Subsequently, any damaged mitochondrion will display a low membrane potential, which can be detected by PINK1 (fly Pink1). PINK1 then phosphorylates and promotes PRKN-mediated ubiquitylation of MFN2 (fly Marf; Mitochondrial assembly regulatory factor), a GTPase and key component of fusion; this in turn facilitates PRKN (fly Park) recruitment to the damaged mitochondrion (Chen and Dorn, 2013; McLelland et al., 2018). PRKN in turn further ubiquitylates MFN2 to promote its localization to the mitochondrial outer-membrane (Rakovic et al., 2011). The now ubiquitylated mitofusins are degraded by the ubiquitin-proteasome system (UPS), assisted by p97 (also known as VCP, fly TER94) (McLelland et al., 2018). This prevents fusion of the dysfunctional mitochondrion with functional ones and breaks the MFN2 mitochondria–endoplasmic reticulum (ER) tether, freeing the damaged mitochondrion to undergo mitophagy. These pathways of fission–fusion and mitophagy are highly conserved between humans and flies (Abtahi et al., 2020; Gegg et al., 2010; Guo, 2012). Indeed, it has been shown in both flies and mammals that PINK1–PRKN is essential for mitophagy, and thus mitochondrial health in a general sense. Here, we present evidence that Pink1–Park-mediated mitochondrial fragmentation affects the kidney filtration system and reduces renal function in a fly model of type 2 diabetes.

**Fig. 7.**
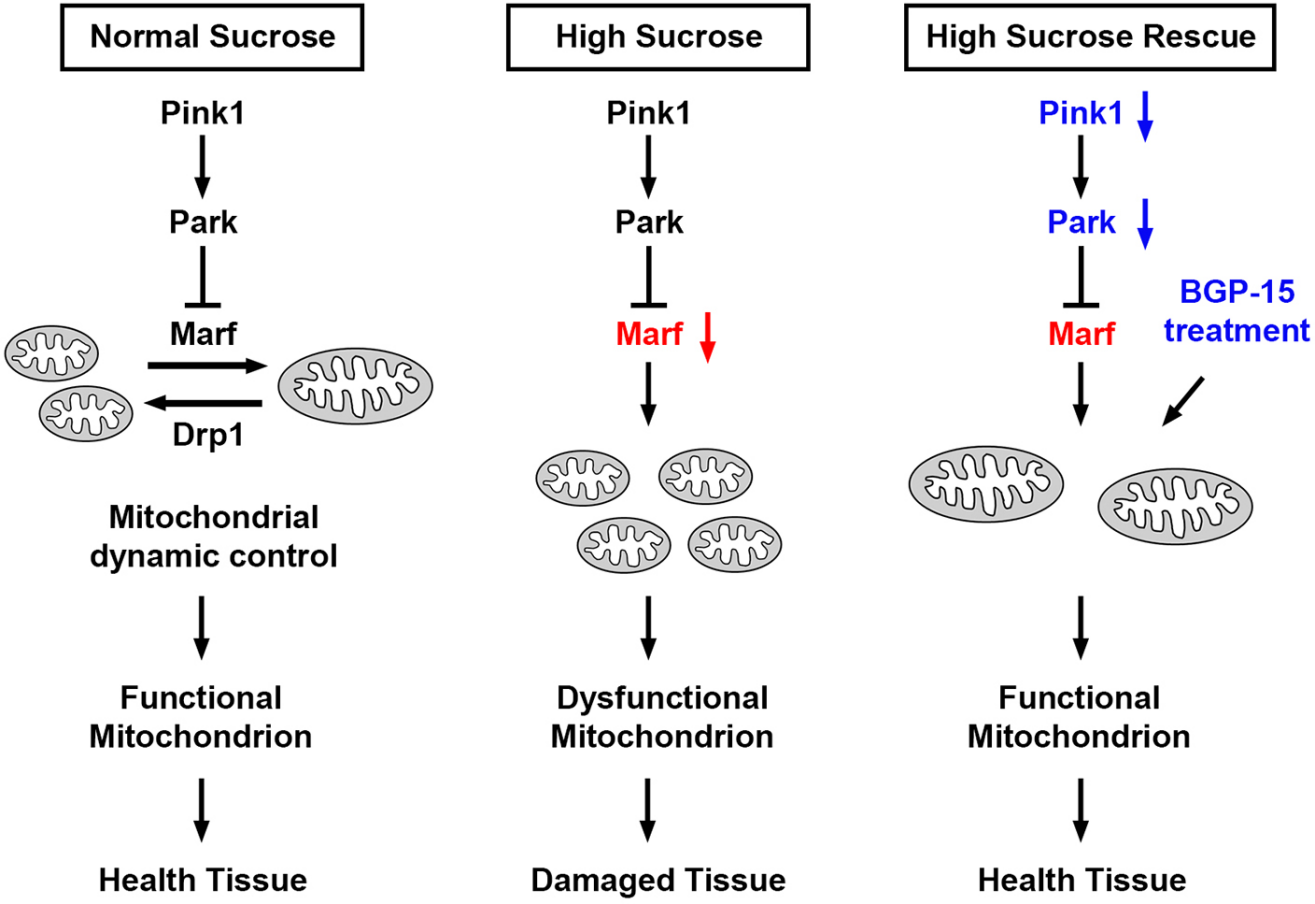
**Model for mitochondrial dynamics-mediated nephrocyte damage caused by high-sucrose, and attenuation through genetic or pharmacological intervention.** Left, schematic representation of simplified mitochondrial dynamic control under a normal diet. Middle, under high-sucrose condition, Pink1–Park pathway-mediated control of mitochondrial dynamics is disrupted, likely mediated by reduced expression of *Marf*, which is associated with diminished mitochondrial fission. These changes in mitochondrial morphology result in mitochondrial dysfunction and tissue damage. Right, both genetic modification of the Pink1–Park pathway, to activate *Marf*, and BGP-15 treatment, can restore mitochondrial dynamics, and thus mitochondrial function, and thereby rescue the nephrocytes.

Mitochondria are the main energy source for the nephrocyte, which makes maintaining a healthy mitochondrial population crucial for its survival. Given that mitochondria are ubiquitous across cell types and tissues, we used nephrocyte-specific expression (*Dot*-Gal4 driver) in all assays. Our data reveal that Pink1–Park-mediated mitochondrial fragmentation causes dysfunctional nephrocyte filtration and renal functional decline under high-sucrose conditions (Fig. 7). Given the highly conserved nature of the pathways involved, these findings likely hold true in humans. In fact, increased renal mitochondrial fission and fragmentation have been shown in patients with diabetic nephropathy (Flemming et al., 2022). Under hyperglycemic conditions, decreased fusion and increased fission have been linked to mitochondrial uncoupling, and the disrupted mitochondrial dynamics have been associated with accumulation of damaged mitochondria, unbalanced ATP levels, increased production of ROS, mitophagy and apoptosis (Chen et al., 2019; Coughlan et al., 2016; Flemming et al., 2022; Kowluru and Mohammad, 2022; Zeng et al., 2019). This clinical picture is recapitulated in our flies when they are subjected to a high-sugar diet or a disrupted Pink1–Park pathway. Like *Marf2* in our flies, studies in human cell culture and animal models have found a disrupted ratio of DRP1 (fission) and MFN2 (fusion) when mitochondrial dynamics are disturbed (Abtahi et al., 2020; Givvimani et al., 2014). In support, renal biopsies from patients with diabetic nephropathy have shown reduced MFN2 (Jiang et al., 2019).

BGP-15 is a nicotinic amidoxime derivate, a bioactive small molecule with chemo- and cyto-protective properties (Pető et al., 2020). Its precise mechanism of action remains unsolved; however, it has been shown to be protective against a variety of conditions, ranging from muscular dystrophy to various cardiac diseases (Pető et al., 2020). BGP-15 was originally developed to treat insulin resistance, which has been demonstrated in animal studies and in a proof-of-concept clinical trial in non-diabetic patients with impaired glucose tolerance (Literáti-Nagy et al., 2009, 2014). *In vitro* and *in vivo* studies of pulmonary hypertension, a mitochondria-related disorder, have demonstrated that treatment with BGP-15 promotes mitochondrial fusion by activating optic atrophy 1 (OPA1). Notably, suppressing MFN2, among other proteins, inhibits BGP-15-induced fusion (Szabo et al., 2018). Other animal studies have shown BGP-15 treatment to be effective in protecting cells against oxidative stress and to extend mitochondrial longevity in a model of heart failure and in Zucker diabetic fatty rats (Horvath et al., 2021; Kozma et al., 2022). Our study expands on these previous findings by providing *in vivo* data that demonstrate that mitochondrial fragmentation leads to renal functional decline that is marked by disrupted filtration, and that this diabetic nephropathy phenotype can be effectively rescued with BGP-15. Unfortunately, a clinical trial for the safety and efficacy of BGP-15 in patients with type 2 diabetes was prematurely withdrawn (NCT01069965). The outcomes of such a trial would be a major first step to establishing the potential of BGP-15 as a therapeutic strategy for diabetes.

## MATERIALS AND METHODS

### *Drosophila* lines

*Drosophila* stocks were obtained from the Bloomington *Drosophila* Stock Center (BDSC; Indiana University Bloomington, IN). The following lines were used in the experiments: UAS-mito-GFP (ID 8442), UAS-*Pink1*-RNAi (ID 31170 and 41671), UAS-*park*-RNAi (ID 31259 and 37509), UAS-*Pink1*-OE (ID 51648), and UAS-*park*-OE (ID 51651). As indicated, two independent RNAi *Drosophila* lines were studied for each gene. The *Dot*-Gal4 (BDSC; ID 6903) driver was used to genetically modify gene expression levels in *Drosophila* nephrocytes. *Hand*-GFP was previously generated by our team (Han and Olson, 2005). Wild-type *w*^1118^ (BDSC; ID 3605) flies were used in the crosses.

### High-sucrose treatment

Sucrose (Sigma-Aldrich) was dissolved in water and added to standard fly food at 0.2 g/ml for the high-sucrose condition. For the normal sucrose condition, water alone was added to standard food. Standard fly food was obtained from Meidi (Meidi, V100) and is based on the BDSC cornmeal food recipe by the Bloomington *Drosophila* Stock Center, which contains ∼7% light corn syrup. Larvae were kept at 25°C to induce transgene expression on normal sucrose and high-sucrose food.

### Dextran uptake assay in *Drosophila* nephrocytes

The dextran uptake assay was used to measure nephrocyte filtration function *ex vivo*. Nephrocytes were dissected from 4-day-old adult flies (females) and kept in artificial hemolymph [70 mmol/l NaCl (Carolina), 5 mmol/l KCl (Sigma), 1.5 mmol/l CaCl_2_·2H_2_O (Sigma-Alrich), 4 mmol/l MgCl_2_ (Sigma-Aldrich), 10 mmol/l NaHCO_3_ (Sigma-Aldrich), 5 mmol/l trehalose (Sigma), 115 mmol/l sucrose (Sigma-Aldrich), and 5 mmol/l HEPES (Sigma-Aldrich), in water]. Cells were incubated with Texas Red-conjugated dextran, 10,000 MW (0.05 mg/ml; Invitrogen) for 20 min, and then fixed with 4% paraformaldehyde in phosphate-buffered saline (1× PBS) (Thermo Fisher Scientific) for 10 min. Dextran uptake capacity was based on nephrocyte fluorescence levels, assayed by fluorescence confocal microscopy (ZEISS LSM 900; see details below). For quantification, 30 nephrocytes from six female adult flies were analyzed per genotype. The results are presented as mean±s.d.

### Quantification of mitochondrial size

To measure mitochondrial size in the nephrocytes, we crossed the *Dot*-Gal4, UAS-mito-GFP fly line with the *Pink1*- and *park*-OE or -RNAi fly lines. Images were obtained with fluorescence confocal microscopy (as detailed above) and processed using ImageJ software (version 1.49). Each individual mitochondrion was manually selected using the Freehand selection function in ImageJ. The mitochondrion area was directly measured by ImageJ. The average size of 30 mitochondria in one nephrocyte was determined. For quantification, 30 nephrocytes in total, obtained from six female adult flies, were analyzed per genotype. The results are presented as mean±s.d.

### Adult survival assay

Following egg-laying, *Drosophila* larvae were kept at 25°C, standard conditions; this temperature is also optimal for UAS-transgene expression. Adult male flies were maintained in vials in groups of 20 or fewer. The number of flies that were alive in each group was recorded every second day. The assay was ended when no survivors were left for any of the lines. 100 flies were assayed per genotype.

### Fluorescent immunochemistry

Adult flies (females) were dissected and heat-fixed for 20 s in 100°C artificial hemolymph [70 mmol/l NaCl (Carolina), 5 mmol/l KCl (Sigma-Aldrich), 1.5 mmol/l CaCl_2_·2H_2_O (Sigma-Alrich), 4 mmol/l MgCl_2_ (Sigma-Aldrich), 10 mmol/l NaHCO_3_ (Sigma-Aldrich), 5 mmol/l trehalose (Sigma-Aldrich), 115 mmol/l sucrose (Sigma-Aldrich), and 5 mmol/l HEPES (Sigma-Aldrich), in water]. Primary mouse monoclonal anti-Pyd antibody (PYD2) (Wen et al., 2020) was obtained from Developmental Studies Hybridoma Bank (DSHB; University of Iowa, IA) and was used at 1:100 dilution in 1× PBS with 0.1% Triton X-100 (Sigma-Aldrich) (PBST). The Alexa Fluor 555-conjugated anti-mouse IgG secondary antibody (A-21422, Thermo Fisher Scientific) was used at 1:1000 dilution in PBST. The nephrocytes were washed with PBST three times, blocked in PBST with 2% bovine serum albumin (BSA; Sigma-Aldrich) for 30 min, incubated with primary antibody at 4°C overnight, washed with 1× PBST three times, incubated with secondary antibody at room temperature for 2 h, washed with 1× PBST three times, and mounted with Vectashield mounting medium (H-1000, Vector Laboratories).

### Fluorescence confocal microscopy

Confocal imaging was performed with a ZEISS LSM 900 microscope using a 63× Plan-Apochromat 1.4 NA oil objective under Airyscan SR mode (ZEN Blue, edition 3.0, acquisition software). For quantitative comparison of intensities, settings were chosen to avoid oversaturation and applied across images for all samples within an assay. ImageJ Software Version 1.49 was used for image processing.

### Mitochondrial membrane potential assay in *Drosophila* nephrocytes

Tetramethylrhodamine, methyl ester (TMRM) is a cationic fluorescent dye that is readily sequestered by active mitochondria. The TMRM assay provides an indication of the mitochondrial membrane potential in live cells (in this case, *ex vivo*). Nephrocytes were dissected from 4-day-old adult flies (females) and kept in artificial hemolymph [70 mmol/l NaCl (Carolina), 5 mmol/l KCl (Sigma-Aldrich), 1.5 mmol/l CaCl_2_.2H_2_O (Sigma-Alrich), 4 mmol/l MgCl_2_ (Sigma-Aldrich), 10 mmol/l NaHCO_3_ (Sigma-Aldrich), 5 mmol/l trehalose (Sigma-Aldrich), 115 mmol/l sucrose (Sigma-Aldrich), and 5 mmol/l HEPES (Sigma-Aldrich), in water]. Cells were incubated with TMRM (1 µg/ml; Invitrogen) for 1 h. Mitochondrial membrane potential was based on TMRM fluorescence levels, assayed by fluorescence confocal microscopy in live cells (ZEISS LSM 900; see above). For quantification, mitochondria in 30 nephrocytes from six female adult flies were analyzed per genotype. The results are presented as mean±s.d.

### ATP measurements

ATP levels in whole flies (females) were measured and normalized using a luciferase-based bioluminescence assay as described previously (Zhu et al., 2021). Each female fly was homogenized in 6 M guanidine-HCl (SRE0066, Sigma-Aldrich) and frozen in liquid nitrogen. Next, samples were boiled for 3 min, cleared by centrifugation at 14,000 ***g*** for 5 min, and diluted 1:10,000 in distilled water (Invitrogen) to measure ATP level (ATP Bioluminescent Assay kit; Sigma-Aldrich) according to the manufacturer's protocol. The colorimetric reaction was measured at 450 nm on a Spark multimode microplate reader (Tecan, Switzerland; SparkControl software, v2.3).

### Quantitative RT-PCR analysis

RNA from nephrocytes dissected from 50 adult flies for each genotype was isolated using TRIzol reagent (Invitrogen). RNA purity and concentration were determined using a Nanodrop-1000 (Thermo Scientific). Total RNA (1 μg) was reverse transcribed using Superscript IV (Invitrogen). SYBR Green based real-time quantitative (q)PCR (Power Cyber Mastermix; Applied Biosystems) was performed on a StepOne Plus machine (Applied Biosystems) using gene-specific primer pairs (Integrated DNA Technologies). Quantitative values were determined using the 2-ΔΔCT method (Livak and Schmittgen, 2001) and normalized to *Gapdh1* as the endogenous reference gene. Values were derived from three qRT-PCR experiments, in each an independent pooled RNA sample was used. Primer sequences were as follows: *Marf*-forward, 5′-AAGCTCTGCGAGAGCAGTTT-3′; *Marf*-reverse, 5′-CGCCTTTGACACCTTCTCCT-3′; *Gapdh1*-forward, 5′-GGCATCGATCTGATCTCGCA-3′; *Gapdh1*-reverse, 5′-GAAGTGGTTCGCCTGGAAGA-3′.

### ROS level assay in *Drosophila* nephrocytes

Dihydroethidium (DHE) is a fluorescent compound used to detect the generation of ROS *ex vivo*; it specifically detects superoxide and hydrogen peroxide. Nephrocytes were dissected from 4-day-old adult flies (females) and kept in artificial hemolymph [70 mmol/l NaCl (Carolina), 5 mmol/l KCl (Sigma-Aldrich), 1.5 mmol/l CaCl_2_·2H_2_O (Sigma-Aldrich), 4 mmol/l MgCl_2_ (Sigma-Aldrich), 10 mmol/l NaHCO_3_ (Sigma-Aldrich), 5 mmol/l trehalose (Sigma-Aldrich), 115 mmol/l sucrose (Sigma-Aldrich), and 5 mmol/l HEPES (Sigma-Aldrich), in water]. Cells were incubated with DHE (1 μg/ml; Invitrogen) for 30 min, and then fixed with 4% paraformaldehyde in 1× PBS for 10 min. DAPI (Invitrogen) was used to label the nucleus at 1:1000 dilution in PBST. The ROS level was based on DHE fluorescence levels, assayed by fluorescence confocal microscopy (ZEISS LSM 900; see above). For quantification, 30 nephrocytes from six female adult flies were analyzed per genotype. The results are presented as mean±s.d.

### Treatment with BGP-15

Compound BGP-15 (SLK-S8370, Selleckchem) was dissolved in water and added to standard or high-sucrose fly food at various concentrations (5, 10 or 20 μM dilution). For controls, water alone (0 mM) was added to the fly food. Flies were treated from the first-instar larva stage.

### Statistical analysis for *Drosophila* assays

Statistical tests were performed using PAST.exe software [Natural History Museum, University of Oslo (UiO), Oslo, Norway]. Data were first tested for normality using the Shapiro–Wilk test (α=0.05). Normally distributed data were analyzed by either a two-tailed unpaired Student's *t*-test (two groups) or by one-way ANOVA followed by a Tukey–Kramer post-test for comparing multiple groups. Non-normal distributed data were analyzed by either a Mann–Whitney *U*-test (two groups) or Kruskal–Wallis H-test followed by a Dunn's test for comparisons between multiple groups. Statistical significance (*) is defined as *P*<0.05.

## Supplementary Material

10.1242/dmm.050471_sup1 Supplementary information
