# Supplementary material for: Promoting mitochondrial dynamics by inhibiting the PINK1–PRKN pathway to relieve diabetic nephropathy
Source: Dis Model Mech. 2024 May 1;17(4):dmm050471. doi: 10.1242/dmm.050471 (PMC11095637; doi:10.1242/dmm.050471)
Supplement: Supplementary information [file dmm-17-050471-s1.pdf]

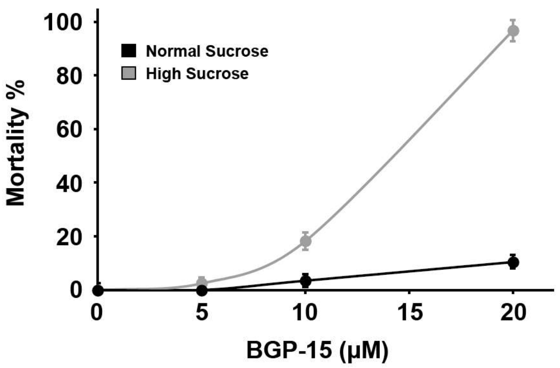

Fig. S1. Dosage-lethality curve for BGP-15 treatment in *Drosophila*

On the X-axis, concentration of BGP-15 in μM; on the Y-axis the percent mortality for normal diet and high-sucrose treated flies (Dot>mito-GFP). n = 4 biological repeats (~50 flies/vial).
